# Evidence of selected nephrotoxic elements in Sri Lankan human autopsy bone samples of patients with CKDu and controls

**DOI:** 10.1186/s12882-020-02049-4

**Published:** 2020-09-03

**Authors:** Thalarabe Bulathge Ananda Jayalal, Sanath Thushara Chamakara Mahawithanage, Senanayaka Mudiyanselage Harshana Mahendra Kumara Senanayaka, Prasanna Bandara Dassanayaka

**Affiliations:** 1 Deputy Director-General Environmental Health Occupational Health and Food safety, Ministry of Health, Nutrition and Indigenous Medicine, Colombo, Sri Lanka; 2 Department of Biochemistry, Faculty of Medical Sciences, University of Sri Jayewardenepura, Colombo, Sri Lanka; 3 Teaching Hospital, Anuradhapura, Sri Lanka; 4 Colombo South Teaching Hospital, Kalubowila, Sri Lanka

**Keywords:** Chronic kidney disease; cadmium, Lead, Mercury, Fluoride, CKDu, CINAC, Nephrotoxins, Sri Lanka

## Abstract

**Background:**

This article describes the analysis and interpretation of data relating to the presence of cadmium, lead, mercury and fluoride in human bone samples obtained from cadavers of patients dying of Chronic Kidney Disease of uncertain aetiology (CKDu) in a case-control study, which the authors believe to be the first in Sri Lanka.

**Methods:**

This is a case-control study comparing selected nephrotoxins levels in autopsy samples of bones from persons confirmed to have died of CKDu, and who had lived in a CKDu hotspot and controls with no history of abnormal kidney functions who had lived in areas having a low prevalence of CKDu.

**Results:**

The average age at death of the cases was 59.6 ± 15.1 (±SD), while that for controls was 58.0 ± 19.3 (±SD) years with no significant statistical difference. Calcium adjusted bone lead and bone fluoride levels were significantly higher among CKDu cases (*n* = 14) than those of controls (*n* = 33). Further, younger and older clusters of CKDu cases can be differentiated when the calcium adjusted lead content was considered. Younger patients reported higher lead content compared to the older group. Cadmium and mercury content did not show a remarkable difference among cases and controls.

**Conclusions:**

Our results show a significant accumulation of lead in bone tissue in persons who have died of CKDu which indicates a higher chronic exposure of the CKDu victims, to lead. Fluoride content in the bones of cases is also significantly high. The results indicate that a gradual reduction of glomerular filtration rate (GFR) may have occurred with the chronic exposure to lead. At a certain point of the threshold of low GFR, excretion of fluoride may be impaired, since the main excretory pathway of fluoride is via the kidneys. Fluoride accumulation in blood in turn may exacerbate nephrotoxicity, triggering a synergistic cascade of events which may lead to a further deterioration in the GFR. The extremely high fluoride content detected suggests a cumulative effect in people exposed to comparatively higher fluoride levels, most probably from drinking water.

## Background

This article describes the analysis and interpretation of the data from a case control study of cadmium, lead, mercury and fluoride content in human bone samples obtained from persons who have died of Chronic Kidney Disease of uncertain aetiology (CKDu), for the first time in Sri Lanka.

It had been identified, in the decade of the 1990-s, that there was an increase in hospital admissions and mortality due to chronic renal failure in the Anuradhapura Teaching Hospital, the main tertiary care hospital in the North Central province of Sri Lanka. In most of those cases, the underlying cause of renal failure was unknown. The conventional known causes of renal failure, i.e. diabetes, hypertension and glomerular nephritis, were not seen in the majority of these cases [1]. A number of other researchers also supported this observation [2, 3] and this has led to the hypothesis that the causation of chronic kidney disease in this community is likely to be due to prevailing environmental factors [1].

The salient pathological lesions of the identified clinical entity were interstitial lymphocyte infiltration (96.7%), tubulitis (81.4%), glomerular sclerosis (81.4%), interstitial fibrosis (88.1%), tubular atrophy (93.2%), and periglomerular fibrosis (42.4%). Further, immunological tests were negative, thereby excluding renal disease of immunological origin [4].

At present, a large of volume credible data is available that supports the presence of the contaminants tested in the study, in various constituents of environmental, food and biological samples in Sri Lanka [5–15]. Among these contaminants, a relatively higher amount of lead has been reported in the food of people living in hotspots of Chronic kidney disease of uncertain aetiology (CKDu) which is also known as Chronic Interstitial Nephritis in Agricultural Communities (CINAC). Lead is found in rice and vegetables, which are the main constituents of the local diet, exposing an individual of 60 kg to an amount of 924.1 μg lead per week based on a model diet of 3 kg of rice and 1 kg of vegetables per week [13].

As rice consumption may be possibly higher than the 3 kg per week among younger people, such exposure may be even higher than the above estimate. According to the latest European Food Safety Agency (EFSA) Panel on contaminants (CONTAM panel) estimations, such exposure is also likely to be associated with a 4.3% reduction of full-scale Intelligence Quotient (IQ), 1.5% annual increase of population-based systolic blood pressure and 36% reduction in Glomerular Filtration rate (GFR) [16] which are serious public health concerns.

Chronic kidney disease of uncertain aetiology (CKDu) shows conspicuous geographical localization in some areas of Sri Lanka (described as hotspots). Most of these are remote agricultural areas of the island. People living in these areas report having food habits that are different from their counterparts living in other areas of the country [13] due to geographical, socio-economic and other factors.

The existence of contamination of food by lead is further corroborated by the blood lead levels detected in exposed people. In a previous study, it had been shown that the study population had had a mean value of 3.35 ± 1.47 μg/dL of lead in their blood [13]. A similar result has been reported in another hotspot (Medirigiriya) with a mean of 3.6 μg/dL [10]. Blood lead levels of CKDu patients living in these hotspots ranged from 1.03 μg/dL to 9.09 μg/dL.

Dietary habits of Sri Lankans, in general, are distinctly different from most of their global counterparts and is included in the G14 cluster of global dietary consumption groups according to the WHO GEMS diet categorizations [17]. Other countries included in this cluster are Comoros, Fiji Islands, Kiribati, Papua New Guinea, Solomon Islands and Vanuatu. A common feature among this cluster of countries is the high consumption of cereals, especially rice, in the diet. This dietary pattern has been confirmed by previous studies [13, 18]. Because of this peculiar food preference and habit, lifelong exposure to lead from the diet is a potential public health problem. Lead is known to enter the food chain through environmental sources and agronomic practices.

Furthermore, the above data raises serious concerns about chronic exposure to lead, starting from the prenatal stage and extending throughout life, since lead is known to cross the placenta and exposure occurs from contaminated food thereafter. It is known that lead is deposited in bone over a person’s lifetime. Bone lead undergoes constant interchange with lead circulating in the blood, as bone remodelling is a physiological process where calcium resorption and deposition occurs along with the accumulated lead. In pregnancy, the fetus receives its calcium via its mother’s blood, and a dose of lead could also be received by the fetus along with calcium [16] should the mother have lead in her system.

Chronic exposure to both cadmium and lead, as well as to lead alone, has been associated with chronic renal failure among other health outcomes [19, 20]. Cadmium and lead exposure through food has been identified as the main mode of exposure in non-occupational settings [21]. Common mechanisms causing nephropathy induced by toxic metals were described by Sabolić [22]. An increased risk of nephropathy was reported in workers with a lead blood level over 62 μg/dL in the1980s [20]. This value was lowered in 2010 by EFSA CONTAM panel following more evidence and technological advances. It has determined the 95th percentile lower confidence limit of the benchmark dose (BMD) of 10% change in the reduction of GFR, (BMDL_10_) as 1.5 μg/dL of blood lead [16].

Chronic low-level exposure to lead and cadmium have been hypothesized as risk factors in not only CKDu but also in neurodevelopmental defects in children, hypertension in adults, neoplasms, low birth weight and malnutrition in various studies [16, 23–29]. Further, the synergistic effect of cadmium and lead on nephrotoxicity has been described in the scientific literature [20, 30]. In addition to lead, exposure to other elements such as cadmium, mercury and fluoride in affected populations appears to be of concern. Probable associations have been shown with the above elements in a number of studies [30, 31]. The above elements have not been previously studied in human biological samples of the affected population adequately.

The best indicator of the chronic exposure of lead is lead content in bones [32]. There is no previous reference or record of data on the lead content in bones for the affected populations. Our study was designed to analyze lead, cadmium, mercury, and fluoride content in autopsy samples of bone along with calcium in deceased persons confirmed to have had CKDu and who lived in CKDu hotspots, and in bone of deceased persons with no history of abnormal kidney functions, who lived in areas having a low prevalence of CKDu.

The association of aging and loss of kidney function has been known for decades. With aging, many subjects exhibit a progressive decrease in glomerular filtration rate (GFR) and renal blood flow (RBF), and with wide variability between individuals. Structural changes, including loss of renal mass; hyalinization of afferent arterioles and in some cases, development of aglomerular arterioles; an increase in the percentage of sclerotic glomeruli; and tubulointerstitial fibrosis are reported. In addition, aging is reported to be associated with altered activity and responsiveness to vasoactive stimuli, such that responses to vasoconstrictor stimuli are enhanced, while vasodilatory responses are impaired. These changes may predispose the older kidney to acute injury as well as to progressive chronic kidney disease. In general, Glomerular Filtration Rate is low at birth as the kidneys are immature. Kidneys develop their full concentrating capacity at the age of two years at which point they gain full filtration power. Thereafter, the filtration power remains constant throughout childhood and early adulthood and starts to decline gradually after the forties [33–35]. Therefore, the age-associated decline of renal functions has to be also considered when interpreting the impact of chronic exposure of nephrotoxins.

## Methods

### Study design

This is a case-control study comparing fluoride, cadmium, mercury and lead levels in autopsy samples of bones between persons with confirmed chronic kidney disease of uncertain aetiology living in an area of high CKDu prevalence and those of deceased persons with no history of abnormal kidney functions living in areas of low CKDu prevalence.

### Study setting

Bone samples were collected from two teaching hospitals where routine autopsies are performed on deceased patients. Bone samples of CKDu patients from the Anuradhapura Teaching hospital, situated in North Central province of Sri Lanka (where CKDu prevalence is the highest) were used. The medical history of the deceased persons was examined and those who were diagnosed with and had died of CKDu were taken as cases.

Autopsy samples from deceased persons from the Colombo South Teaching Hospital, Kalubowila, were collected as controls. The Colombo South Teaching Hospital is situated in the Western province of Sri Lanka, where the CKDu prevalence is known to be low. The medical history of deceased persons from the Colombo South Teaching Hospital, Kalubowila was examined and those with any history of renal disease were excluded from the study.

### Sample collection

Samples were collected after obtaining the permission of the next of kin of the deceased persons who were subjected to autopsy. The autopsies were conducted for reasons other than this study, such as for example the confirmation of the cause of death for a variety of statutory requirements. An information sheet in the preferred language was given to the next of kin. With written consent secured, a piece of sixth right rib from the midclavicular line was obtained.

Morphological appearances of kidney were recorded at the autopsy examination for subsequent analysis.

Samples were stored in refrigerated conditions soon after the collection and later airfreighted to the Laboratory of Pathophysiology, University of Antwerp, Belgium under refrigerated conditions with dry ice. Information about the samples such as patient details, cause of death, whether they were cases or controls were not disclosed to the laboratory.

Bone samples were wet weighed. For the analysis, pieces of bone weighing between 50 and 300 mg were placed in a CEM-Discover SP microwave synthesizer and digested in a ultrapure concentrated HNO_3_ – H_2_O_2_ mixture. Following digestion, samples were adjusted to 10 mL with ultrapure demineralized Milli-Q water, thereby bringing the acid concentration in the solutions to approximately 2%.

Cadmium, lead, mercury and calcium were measured in the digestion liquid by means of high resolution inductively coupled plasma mass spectrometric analysis HR-ICP-MS (Element XR, Thermo Scientific, Finnigan Element 2, Bremen, Germany). In order to overcome matrix interferences, cadmium, lead, mercury and calcium concentrations were accurately quantified by applying the standard addition calibration technique, whereby a series of aliquot sample solutions were spiked with increasing concentrations of the respective elements. If measured values exceeded the calibration range for an analyte, they were diluted and reanalyzed. Method validation was achieved by analysis of a biological tissue reference material BCR 668 (European Commission – Joint Research Centre, Geel, Belgium) which was processed in a similar manner as the tissue samples. During analysis, a National Institute of Standards and Technology (NIST) reference standard (SRM1640a, USA NIST, Gaithersburg, USA) was used as analytical quality control (QC) check. For each of the elements under study, 4 aliquots were analysed, yielding respective concentrations of 3.997, 3.928, 4.021 and 3.966 μg/L for cadmium (reference value 3.922 μg/L), 12.045, 12.039, 12.148 and 12.011 μg/L for lead (reference value 12.101 μg/L), 0.196, 0.197, 0.196 and 0.199 μg/L for mercury (reference value 0.200 μg/L) and 5580.4, 5499.8, 5670.4, 5524.9 μg/L for calcium (reference va1ue 5615.0 μg/L) respectively. Meticulous care was taken to avoid external contamination through reagents and materials. Blank values were tested, and results were 0.001 μg/L for cadmium, 0.004 μg/L for lead, 0.002 μg/L for mercury and 1.1 μg/L for calcium respectively.

Limits of Quantification (LOQs) were set at 0.05 μg/L, 0.01 μg/L, 0.01 μg/L and 3 μg/L for cadmium, lead, mercury and calcium respectively.

Coefficients of variation (CV) for measurement were calculated by doing ten sequential within-run measurements of a sample with intermediate concentration. Coefficients of variation for measurement of the elements under study in the digestion liquids were 1.0, 0.5, 0.7 and 1.4% for cadmium, lead, mercury and calcium respectively.

In order to check for within tissue heterogeneity, duplicate parts were taken in 25 bone samples and the average ± Standard Error of Mean (SEM) difference between the two parts for all samples was calculated yielding values of 27.7 ± 4.8% for cadmium, 19.8 ± 3.0% for lead, 31.3 ± 5.9% for mercury and 21.4 ± 3.3% for calcium respectively. Since lead is mainly deposited in bone with calcium, the variation could substantially be reduced by calculating the lead/calcium ratio (i.e. correction for bone density) yielding a value of 13.2 ± 2.9%.

For fluoride measurement, a colorimetric method was used. Fluoride is measured in the same digestion liquids as for the other elements under study by means of the Machery-Nagel colorimetric kit (NANOCOLOR Test 0–40, Fluorid 2; Düren, Germany). With this method, fluoride shifts the color of the lanthanum-alizarin complex to violet, after which the measurement is performed photometrically at 620 nm. With this method, solutions with fluoride content varying between 0.1–2.0 mg/L can be measured. No standard reference material for measurement of fluoride in tissues is available.

All statistical analyses were performed by Excel and SPSS ver. 23.0. Differences of levels of nephrotoxic elements between cases with CKDu vs. controls were compared. Associations between age at death and levels of nephrotoxic elements were explored with regression analysis and curve fitting was performed to identify the model that provides the best-fit-lines [36]. The behaviour of the best fit lines was studied. The distribution of each variable was checked for normality (Shapiro-Wilk test), and appropriate parametric or non-parametric tests were performed to compare between groups. The Mann-Whitney U test, a non-parametric test was performed to compare two independent means and z statistic was calculated.

Geographic distribution of cases and controls was illustrated in a map of Sri Lanka and the map was created using QGIS 3.12.2, an open-source Geographic Information System (GIS) software.

### Ethical considerations

The protocol was approved by the Ethics Committee of the Sri Lanka Medical Association (ERC 17/014).

## Results

### Distribution of geographical areas where the deceased lived

**Fig. 1:**
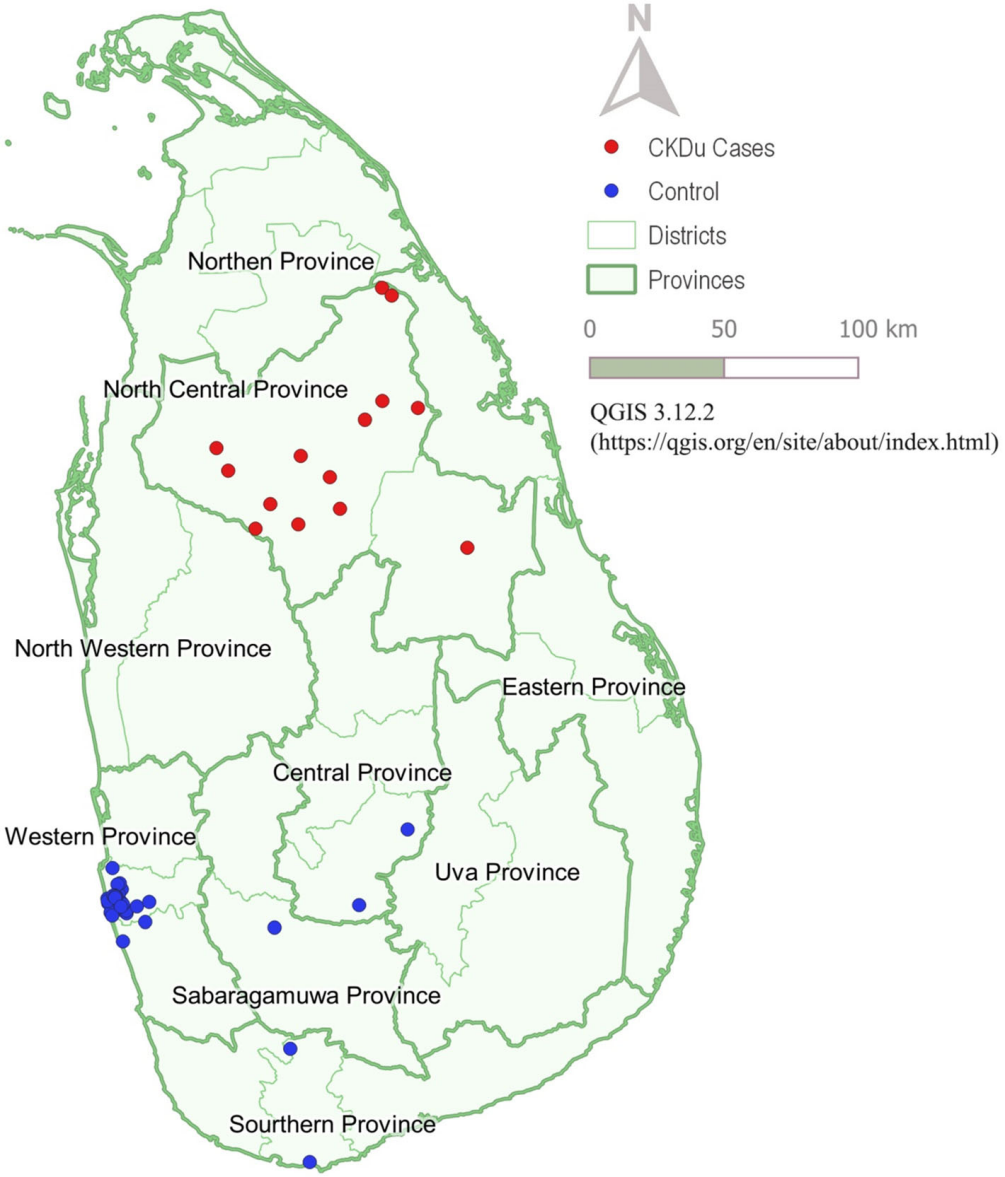
Geographical distribution of cases and controls (The map was created using QGIS 3.12.2)

A sample of 14 cases with CKDu and 33 controls was analyzed. Information on location of residence during the twenty years prior to death and before was obtained, and the geographical distribution of cases and controls is depicted in Fig. 1. Thirteen patients with CKDu were from the Anuradhapura District and one patient from the adjoining district of Polonnaruwa, thereby establishing that all cases with CKDu were from the North Central Province. Twenty-eight controls were from western province [Table 1 and Fig. 1], two each from the Nuwara Eliya and Matara Districts, and one from the Ratnapura district. All controls therefore, were from areas of low CKDu prevalence.

**Table 1:** Geographical distribution of CKDu patients and controls

Province	District	Cases with CKDu	Controls	Total
North Central	Anuradhapura	13	0	14
	Polonnaruwa	1	0	
Western	Colombo	0	28	33
Southern	Matara	0	2	
Sabaragamuwa	Ratnapura	0	1	
Central	Nuwara Eliya	0	2	

There were more males among the CKDu cases as compared with the non-CKDu controls, but the difference was not statistically significant (Table 2). Similarly, there was no statistically significant difference in the age at death between patients with CKDu (59.6 ± 15.1 years) and controls (58.0 ± 19.3 years) (Z = -0.454; *p* = 0.650).

**Table 2:** Distribution of males and females

Gender	non CKDu		CKDu	
	n	%	N	%
Male	22	66.7	12	85.7
Female	11	33.3	2	14.3
Total	33	100.0	14	100.0

Fisher’s Exact Test *p*-value = 0.288

Out of fourteen positive CKDu cases, none had been diagnosed with diabetes prior to death. Nine had reported no hypertension. Two had hypertension prior to the diagnosis of renal failure, and one person was detected as being hypertensive at the time of diagnosis of renal failure. Two individuals were diagnosed as hypertensive two years after the diagnosis of renal failure. Macroscopic changes of the kidneys of twelve cases were observed as contracted small kidneys with a granular surface, consistent with the general features of CKDu. Even though granular smaller kidneys are not specific for CKDu per se, CKDu is one of the commonest reasons for granularity and contraction leading to smaller kidneys in autopsy findings in the endemic areas. Two cases had normal sized kidneys with a granular surface, which probably indicates early pathology. In contrast, 27 controls had normal size kidneys, 17 of them presenting a smooth surface, and 10 presenting a granular surface, of which six showed granular contracted kidneys. However, none of the controls showed any evidence of kidney disease prior to death.

Lead, calcium, calcium adjusted lead, cadmium and fluoride content of bone samples of CKDu cases and the controls are given in Table 3.

**Table 3:** Lead, calcium, lead per unit calcium, cadmium and fluoride content of bone samples of CKDu cases vs. controls

Variable	CKDu (*n* = 14)		controls (*n* = 33)		z value*	*p* value
	Mean	SD	Mean	SD		
Lead μg/g	1.09	0.80	0.66	0.42	−1.815	0.070
Calcium μg/g	70,740.32	30,713.50	72,081.67	46,981.64	−0.837	0.402
Calcium adjusted lead ×10^−6^	14.68	7.51	10.38	5.74	−2.256	0.024
Cadmium μg/g	0.030	0.037	0.036	0.042	−0.782	0.434
Mercury μg/g	0.002	0.003	0.002	0.003	0.000	1.000
Fluoride μg/g	594.16	335.82	22.49	44.50	−5.719	< 0.001

* As the dataset was not normally distributed, the non-parametric test (Mann-Whitney U test) was performed to compare the two means and the z statistic was calculated by the Mann-Whitney U test

The distribution of each variable was checked for normality (Shapiro-Wilk test) and found to be not normally distributed (*p* < 0.001 for all variables). Hence the Mann-Whitney U test was performed.

Calcium adjusted lead content was 41% higher in the CKDu cases than in the controls and the difference was statistically significant (*p* = 0.024). Absolute lead content was also 65 % (65%) higher in the CKDu cases than in the controls, though the difference was not statistically significant (*p* = 0.070).

Fluoride was found to be 2542% higher in CKDu cases than in the controls, showing a statistically significant difference (p < 0.001). However, there were no significant differences in calcium, cadmium and mercury content between the two groups.

Age at death of the study population ranged from 23 to 90 years with a mean of 58.5 ± 18.0 years.

**Fig. 2:**
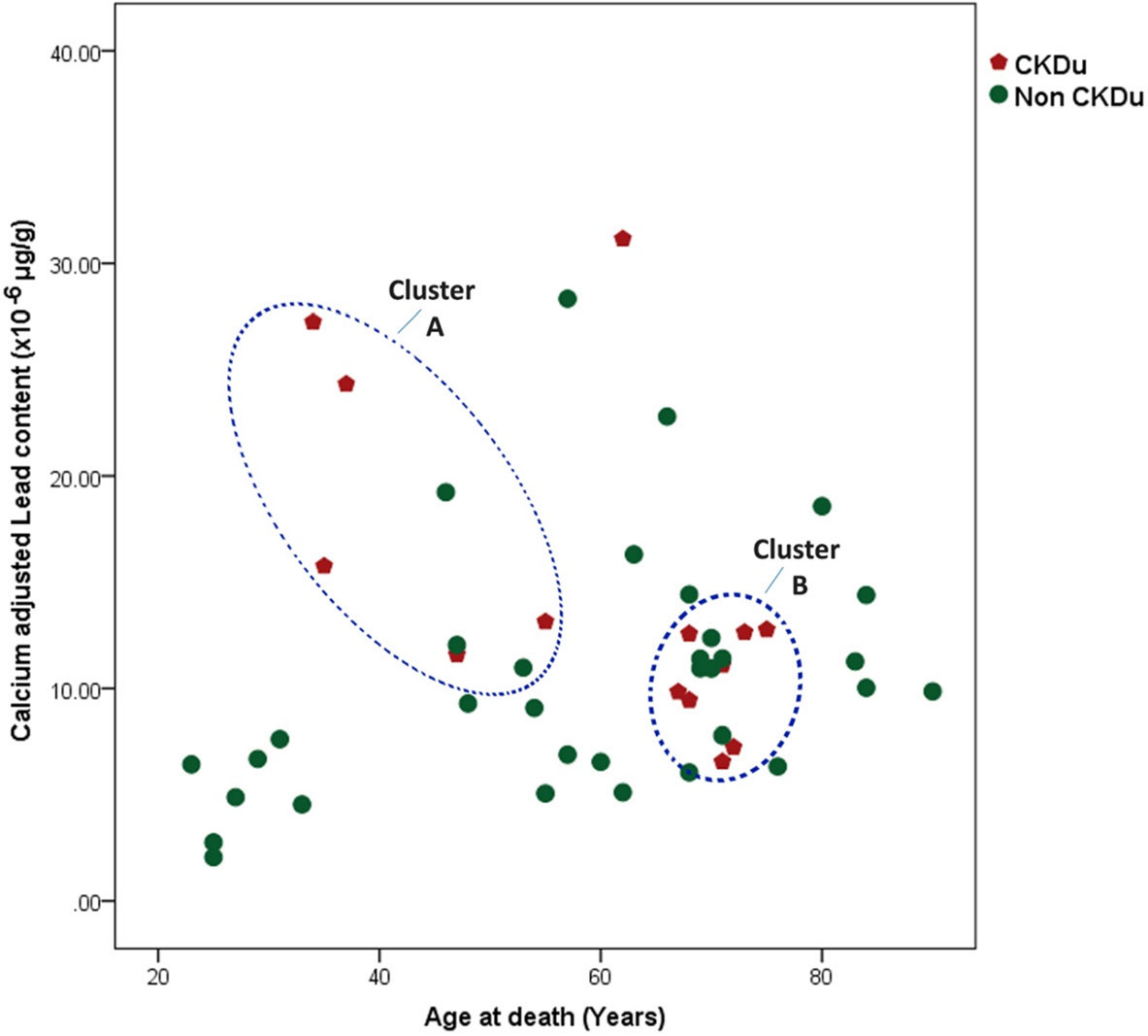
Distribution of calcium adjusted lead content by the age at death of study population. (The two clusters of cases are encircled; Cluster A- Age less than 55 years and higher bioaccumulation of lead; Cluster B Age more than 55 years and lower bioaccumulation of lead)

**Fig. 3:**
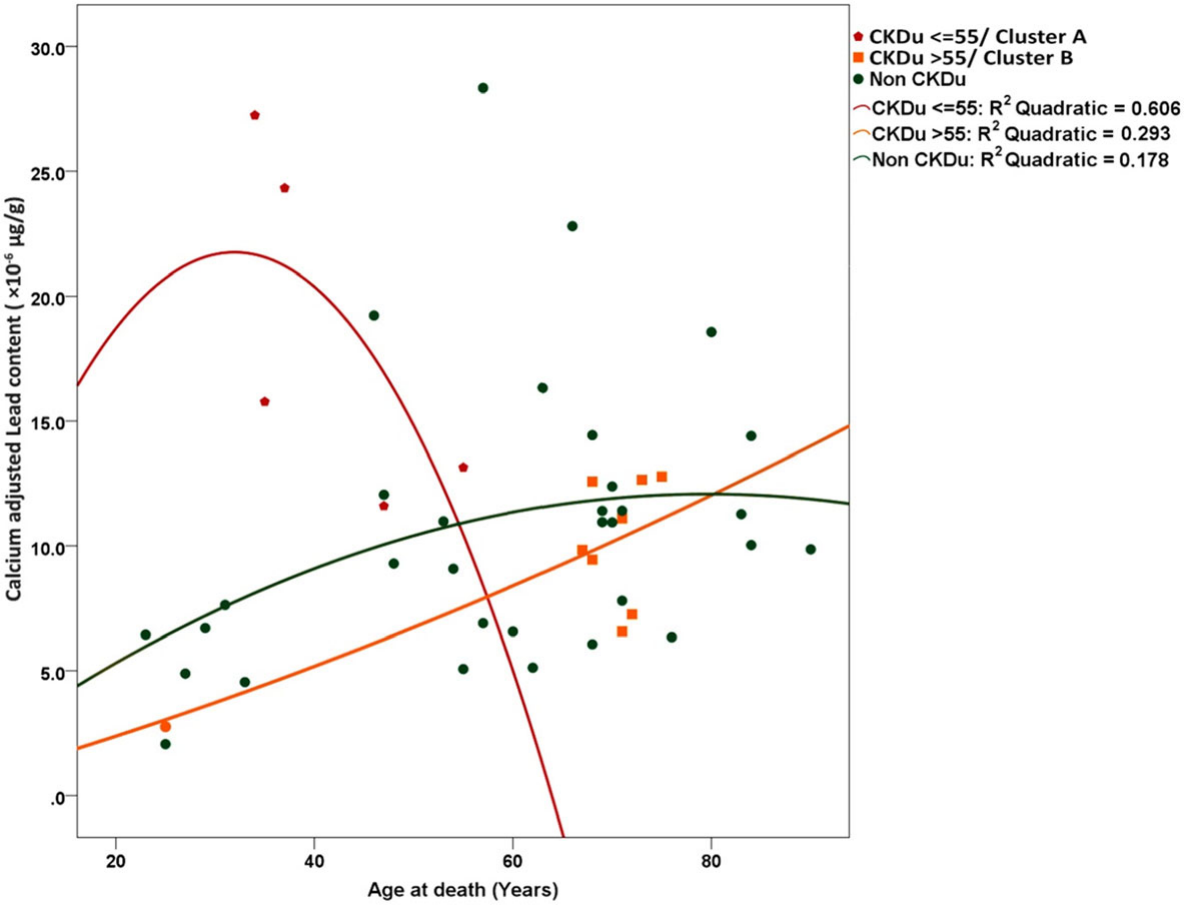
Association between lead content per unit calcium and age at death of study population. Quadratic regression analysis was carried out to generate best-fit lines as they result in higher R^2^ values than linear regression in the three series of data. The cluster A (younger and high in bioaccumulation) (red line) shows a relationship of inverse U appearance between age and calcium adjusted lead levels as they had a shorter life span with higher lead accumulation. Cluster B showed a low level of bioaccumulation (orange line) as compared to cluster A but with a higher level of bioaccumulation than the controls (green line)

Association between the content of selected nephrotoxic elements and age at death of the study subjects was further explored. Considering the calcium adjusted bone lead content at death in CKDu cases, there was an apparent clustering effect, as illustrated in Fig. 2. One case sample from a person aged 62y at the time of death whose calcium adjusted bone lead content of 31.6, did not fit into either cluster for the reason of higher lead content and higher age at death. Therefore, this was considered as an outlier and further analysis was done excluding this specific case. There were two clusters of CKDu cases (encircled in blue in Fig. 2), whereas the controls showed no such pattern and the corresponding values were scattered over a wide range. The two observed clusters may be viewed as two distinct groups on either side of a threshold of age at death of 55y. Accordingly, cases with CKDu who had died at age equal to or below 55 years (cluster A) showed a higher calcium adjusted lead content (*n* = 5; mean = 18.42 × 10^− 6^ ± 7.0) than those with CKDu who died at age above 55 years (*n* = 8; mean = 10.28 × 10^− 6^ ± 2.4, Cluster B). The observed difference was statistically significant. (*p* = 0.0013; Mann Whitney U test). Associations between calcium adjusted lead content at death of the two clusters of CKDu cases and controls were further explored using regression analysis (Fig. 3). Curve fitting was performed to identify the model that provides the best-fit-lines to the three series of data [36]. R^2^ values for best-fit-lines derived by the quadratic model were higher than those derived by the linear model (data not shown) hence the quadratic regression analysis was performed. The behaviour of the best fit lines of the three groups was different from each other. Those with CKDu who died at an age equal to or below 55 years, reported a higher calcium adjusted lead content and distribution within a broader range as compared to the other two groups. Furthermore, they demonstrated an inverse “U” shape of association between the lead content per unit calcium and the age at death indicating they have had accumulated a higher level of lead content during a shorter period. In contrast, those with CKDu who died at the age of above 55 years demonstrated a relatively a linear association, indicating that the process of accumulation of lead had continued for a more extended period. Controls also showed a similar trend. According to the R^2^ values, 60% of the age at death (the response variable) is explained by the lead content among those who died at age equal to or less than 55 years old, whereas corresponding values for CKDu cases who died at the age above 55 years and controls were 29 and 18% respectively.

**Fig. 4:**
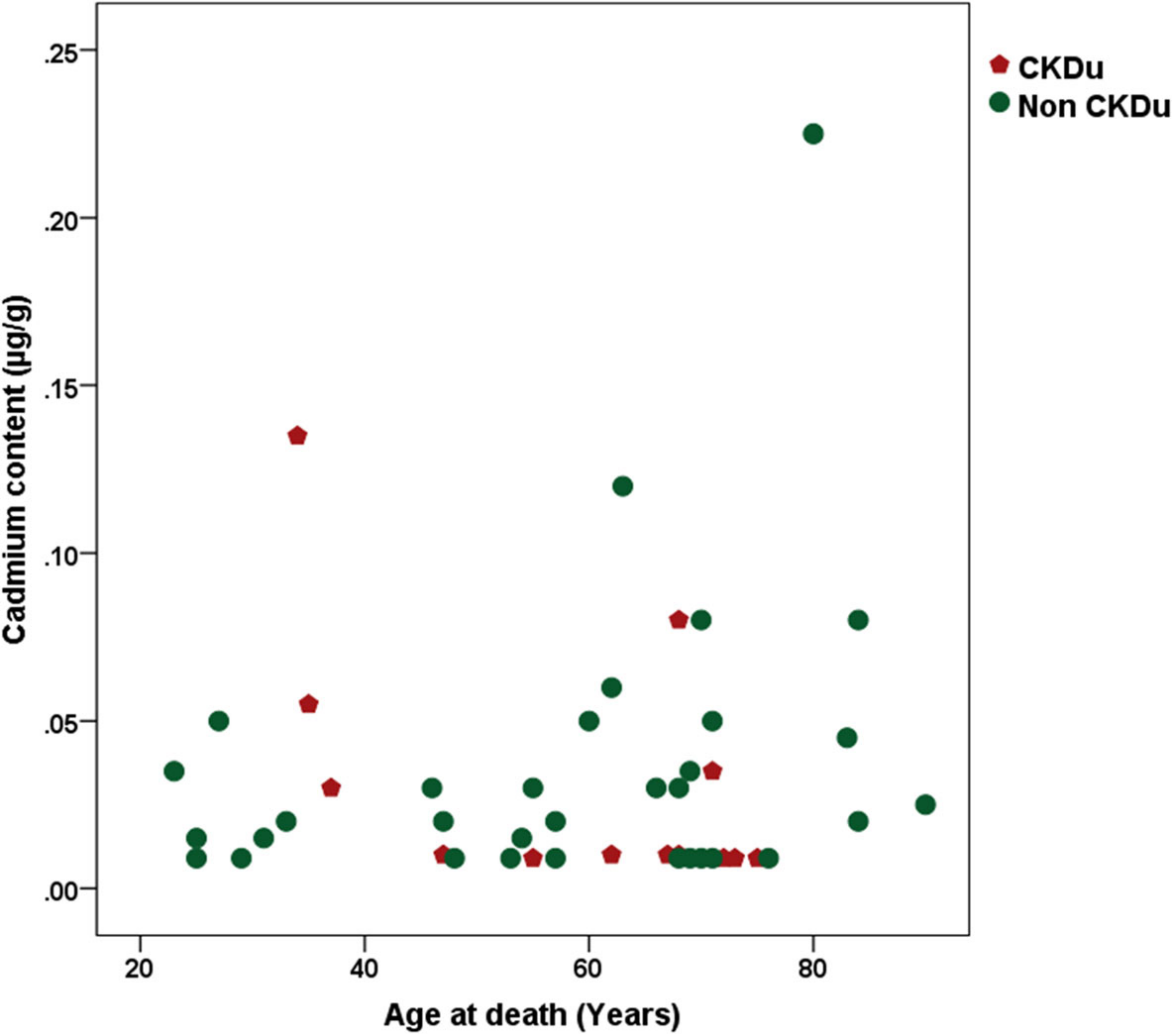
Distribution of cadmium content by age at death of the study population

The association between cadmium concentration and age at death of the study population was explored (Fig. 4). There was no conspicuous clustering apparent as seen for the lead content.

**Fig. 5:**
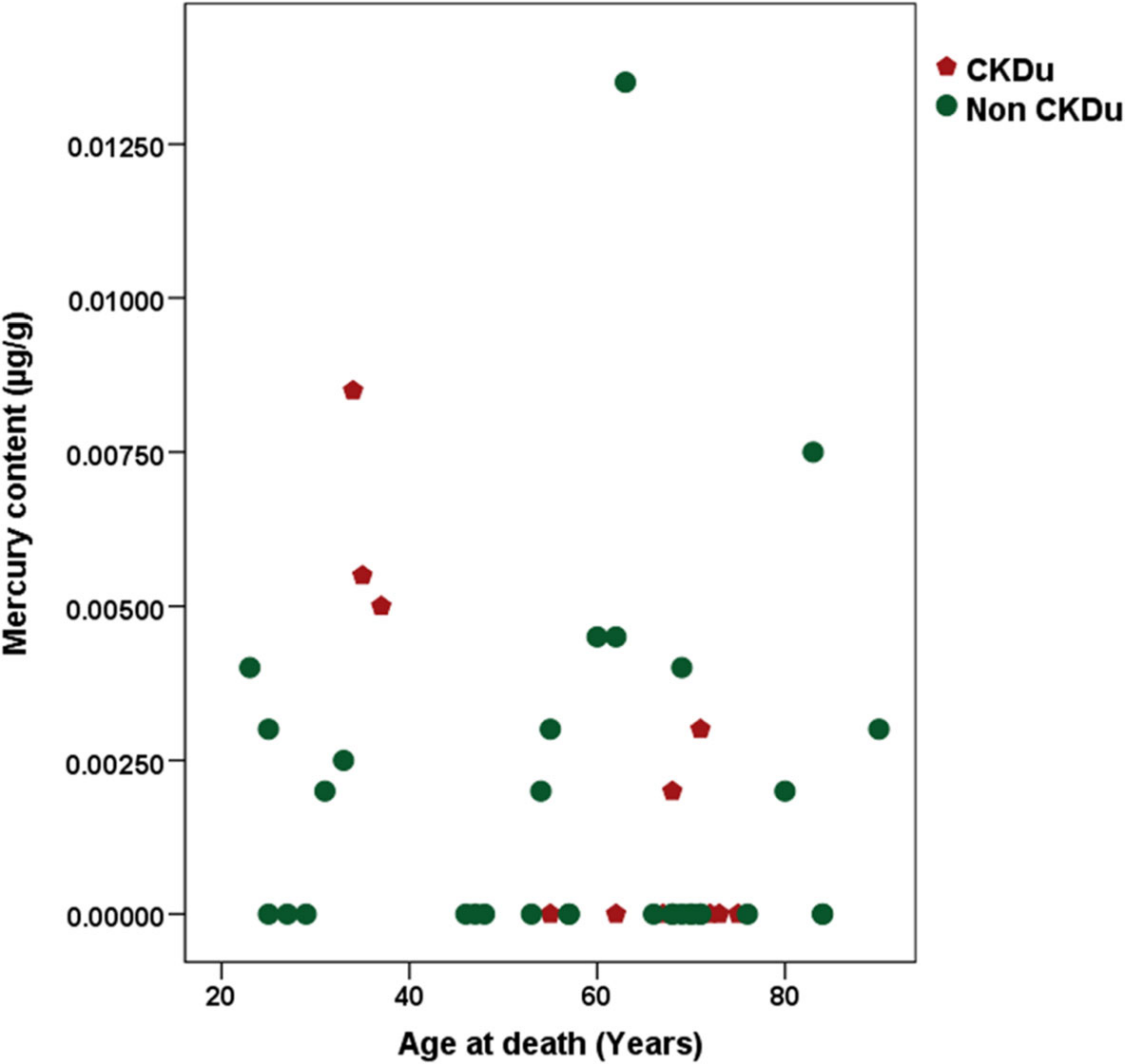
Distribution of mercury content by age at death of the study population

A similar exercise was also done for mercury (Fig. 5). Three of the younger CKDu cases were found to have the highest level of mercury concentration in bone and two controls of the older age group were found to be in the same mercury concentration range as the younger CKDu cases.

**Fig. 6:**
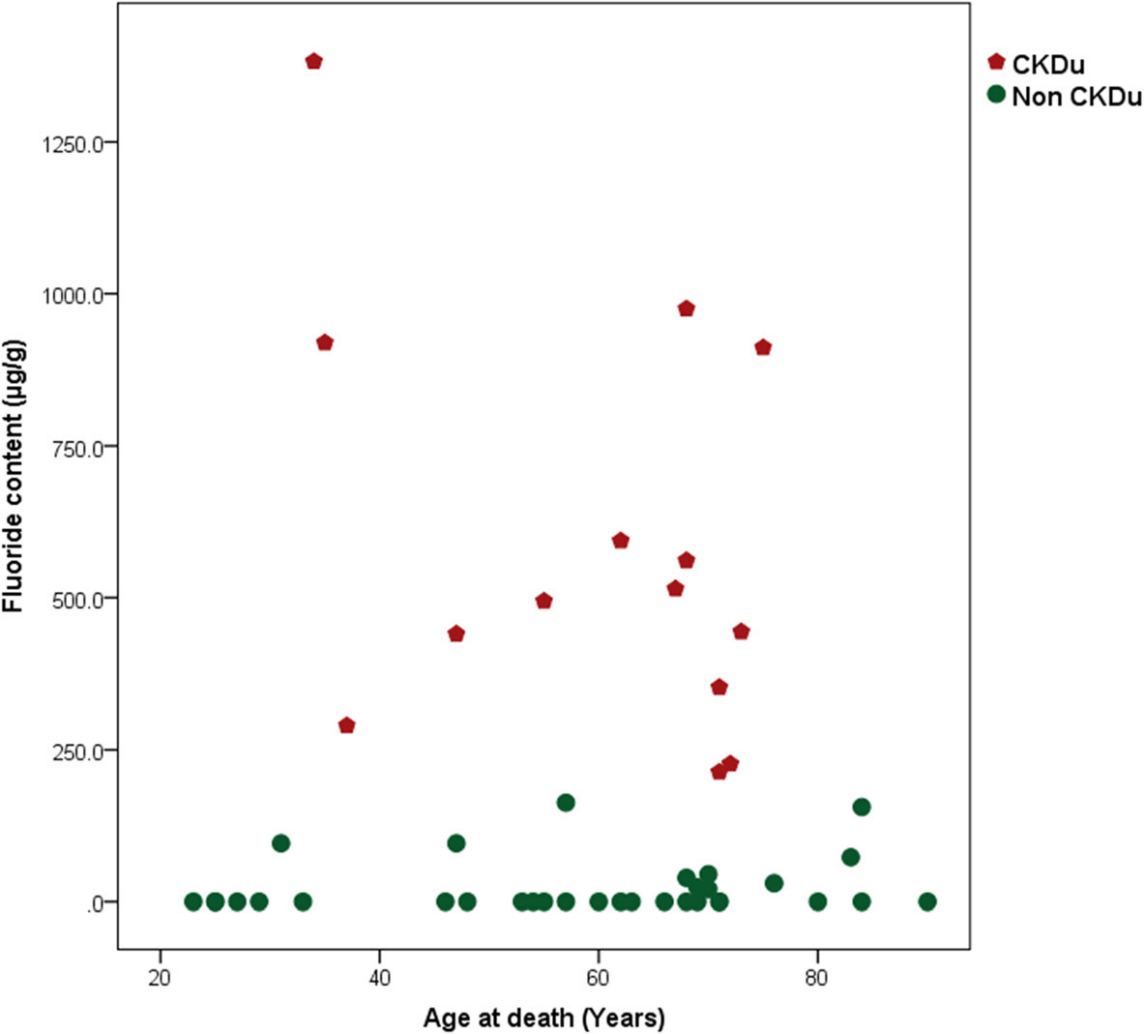
Distribution of fluoride content by the age at death of the study population

Bone fluoride concentration was also subjected to the same analysis (Fig. 6). As expected, no clustering effects within the CKDu cases were visible, but all the CKDu cases showed markedly higher fluoride concentration in their bones.

## Discussion

The average age at death ± SD of the CKDu group was 59.6 ± 15.1 years, indicating a significant reduction of life span in those afflicted with CKDu. In Sri Lanka, life expectancy at birth was 75 years in 2018 and higher among the young adult population. Therefore, this disease can be considered as a cause of premature death and is a significant public health, social and economic concern. The age of death of the control cohort does not give any interpretable information as it is a diverse group having multiple other causes of death.

Kidney morphology of cases was that of chronic renal disease. In contrast, a group of six out of 33 controls representing an older group, showed granular, small kidneys, probably due to undiagnosed mild to moderate renal disease of standard causes. As they had been living in areas of low CKDu prevalence (non-endemic areas), their kidney pathology may be assumed to be unrelated to that of CKDu. The other twenty eight controls had normal kidneys at autopsy.

Bone lead to calcium ratio is known as the best biomarker of chronic lead exposure. Calcium as denominator excludes the effect of metabolic stage of bone and osteopenia or osteosclerosis in the individuals. Lead once absorbed into the blood remains there for a short period and then deposits in bone, as the body handles lead similar to calcium. The half-life of lead in bones of the human body is approximately 10 to 30 years [21]. Therefore, in low dose chronic exposure as described in the study population, bioaccumulation of absorbed lead progresses until about the fifth decade of life, and thereafter an equilibrium of absorption and excretion will be reached. Thus, maximum lead deposition in the bones of the study population is expected in the sixth decade. In a non-occupational setting, it is food that mainly contributes to lead exposure. Contamination from leaded paint may contribute in some settings [37].

There is a constant exchange of calcium between bone and blood as bone resorption by osteoclasts and ossification by osteoblasts is a lifelong process. This calcium exchange releases deposited lead to the bloodstream. This physiological phenomenon is enhanced during pregnancy and may result in higher lead concentration in blood. As lead is known to cross the placenta, the fetus is also likely to be exposed to the harmful effects of lead.

Agricultural soil, fertilizers, and food grown in CKDu hotspots have been reported to be considerably contaminated with lead. This has been reflected further by blood lead levels detected in the exposed population. Blood lead levels from 1.03 to 9.09 μg/dL are reported from the affected population [10, 13]. The present study further confirms that people who died of CKDu have significantly more lead deposited in their bones than the controls, who had no history of abnormal kidney function. As lead is known to cause tubular interstitial nephropathy, we postulate that this association may be causal.

As shown in Fig. 3 in the control group, lead concentration shows a gradual build-up with age indicating exposure of very low dose chronic exposure throughout life in the control population. As lead has a long half-life in the bone, this phenomenon is expected. Further, it can be argued that exposure has occurred from early life and continued throughout indicating exposure to environmental contaminants, possibly from food or air. In contrast, CKDu cases showed two distinct subgroups, i.e. those who died before the age of 55, and those who died after 55y. The former group (where death occurred at 55 years of age or earlier) had accumulated lead in their bones to the highest levels detected (red line). Three out of five cases had succumbed to the disease at ages 34, 35 and 37y respectively. The other two cases were 47y and 55y at the time of death. There is an older subgroup whose death had occurred between 62 and 75 years of age. This older age group demonstrates an accumulation of lower lead levels in their bones than their younger deceased counterparts, and which are probably equal to the controls whose kidney functions were presumably to be largely normal (lead calcium ratio of 6.57 to 16.192). It is known that with the natural process of aging, kidney function starts to deteriorate from the fifth decade of life [33–35]. Therefore, the aging kidney may be more vulnerable to ill effects of lead and may have caused malfunctioning of the kidney at a lower level of exposure.

Our study results show high levels of fluoride in bones of CKDu cases. This may be an effect of low renal function or renal failure, as the main excretory pathway of fluoride is via the kidneys. The failing kidney may cause fluoride to accumulate in the blood and this may result in further deterioration of kidney function [31] as high levels of fluoride cause nephrotoxicity. It may be argued that a slow deterioration of GFR occurs in patients exposed to lead, and at a certain threshold point the kidney is unable to excrete fluoride adequately, resulting in fluoride levels building up. Fluoride intake is known to be higher in CKDu prevalent areas [15]. Therefore fluoride accumulation may initiate a cycle of events that leads to accelerating renal failure. This may explain the pathogenesis of CKDu. The clinical picture of CKDu cases described in Sri Lanka [38] also shows that a person who is otherwise well, falls ill abruptly, and a deterioration of renal function can set in fairly rapidly. In most instances, a patient’s condition can deteriorate to a terminal stage of renal failure within one year. Conventional causes of renal failure due to hypertension and diabetes are not known to show such a rapid deterioration in general.

The other inference that can be drawn is the older age subgroup has probably spent the first 30 years of life in a less toxic environment where lead exposure is less, and with higher exposure to lead arising in the latter years of life. Most of the cases studied are known to have changed their residence around this age (30 years). Further, the environmental concentration of lead in soil, air etc. may have increased during this time too, leading to increased exposure. This observation is compatible with the appearance of CKDu from the 1990-s. It may be argued that environmental lead levels, especially in the food chain, have increased over time, probably due to the use of chemical fertilizer which exceeded the threshold of safe lead levels starting around 30 years ago. In the decade of the 1990’s, Sri Lanka commenced a programme of intense agricultural practices and shifted towards the extensive use of chemical fertilizers [39]. This could have resulted in lead being added excessively albeit unintentionally to soil and thus entered the food chain of inhabitants in those areas, thereby exposing those living at that time in vulnerable areas to a fairly high dose of lead. This observation describes the chronological appearance of CKDu and hence indirectly supports the causal association.

It is possible that the older subgroup of CKDu cases was also exposed to higher fluoride than their control counterparts, making them more vulnerable to renal damage from lead and fluoride combination. Higher fluoride exposure through drinking water and food cooked in this water have being reported in areas of CKDu prevalence.

It may be argued that retention of lead in the bone is an effect of renal failure rather than a causal association. We would argue instead, that the cases studied did not survive sufficiently long enough to build up bone stores of lead to the levels detected after the development of renal disease. The estimated exposures are very low and probably occurred during the lifetime of the individuals, and when the long half-life of lead is considered, lead in the bone is highly unlikely to be an effect of renal failure.

The previous findings of lead in food in a CKD hotspot, and blood lead levels in people living in CKDu hot spots [10, 13] are reaffirmed by the finding of a significant amount of bone lead in persons afflicted with CKDu. Cadmium and mercury content at the time of death were largely similar between the cases and controls, and did not show any observable clustering effect. Even though bone is not considered to be the best tissue to demonstrate exposure to cadmium and mercury in humans, the availability of a control group for comparison, allows our inferences to be drawn with some certainty.

Even though mercury did not show any statistically significant difference between cases and controls, the three youngest CKDu cases who had more intense disease were found to have higher accumulated levels of mercury in their bone at the time of death. This finding could be considered as a basis for further study.

Accordingly, the detected evidence of lead exposure in possible synergy with higher fluoride exposure may describe the causation of chronic kidney disease where the cause cannot be attributed to hypertension, diabetes, glomerular nephritis and other forms of CKD. The phenomenon of fluoride being associated with increased absorption of lead may also serve to exacerbate the effects of lead in this situation [21].

In addition to renal disease, lead exposure may have other negative health outcomes. The European Food Safety Agency (EFSA) Panel on Contaminants in the Food Chain (CONTAM) considers developmental neurotoxicity, systolic blood pressure increase and chronic kidney disease as the most potent adverse effects of lead [16]. Lead is known to cross the placenta and affect the neurodevelopment of the fetus. Bone lead may provide a storage site which continues to release lead to blood in pregnant women, and the developing fetus may be consequently be adversely affected. Neurodevelopmental impairment is the most important effect of lead in the developing fetus and in children, which may lead to permanent cognitive impairment in the affected population.

Accordingly, elevated systolic blood pressure and associated cardiovascular events may also be a serious public health concern in affected populations.

The findings of this study are generalizable to Sri Lanka, as cases were distributed mainly in the North Central Province, while controls were from the Western Province of Sri Lanka.

Histological studies of the specimens of kidneys were not carried out due to resource constraints, which is a limitation of the study. However, this limitation was overcome by taking a comprehensive medical history of the diagnosis of CKDu as the case definition. This was further supported by proxy evidence of the cases having lived in an endemic area, as well as by the observed morphological features of the kidneys at autopsy. Similarly, the control cohort did not have a history of renal disease, lived in non-endemic areas and most of whom had morphologically normal kidneys.

It is recommended that steps be taken to reduce exposure to lead and fluoride in the people living in affected areas. This might be achievable by selectively increasing the proportion of types of food which are less contaminated with lead, such as meat from animal sources and fish; both of which are believed to be less contaminated with lead. Provision of water with a lower concentration of fluoride may reduce both the absorption of lead and further deterioration of renal functions. Further research on how to reduce the contaminants in the food chain is required. Continuous monitoring of lead with other potential contaminants in the environmental and biological samples should be undertaken. Effective advocacy programmes aimed at modifying the dietary habits of people living in the area should be considered necessary in light of this study.

## Conclusions

We have shown a significant accumulation of lead in bones among cases with CKDu as compared with their control counterparts, which indicates higher chronic exposure of cases to lead. Fluoride content in the bones of cases is significantly higher than in controls. There were no significant differences in cases vs. controls with regard to the other nephrotoxic elements studied.

Gradual reduction of glomerular filtration rate may occur with chronic exposure to lead. At a certain point of a threshold of low glomerular filtration rate, excretion of fluoride may be impaired as the main excretory pathway of fluoride is through the kidneys. Fluoride accumulation in blood in turn may lead to nephrotoxicity, triggering a cascade of events leading to a further decrease in GFR. The extremely high level of fluoride observed, i.e. 2542% higher in CKDu patients’ bones compared with controls, suggests a cumulative effect in people exposed to comparatively higher fluoride levels from drinking water.

## Data Availability

The datasets generated and/or analysed during the current study are not publicly available due to the personal nature of information contained therein but are available from the corresponding author upon reasonable request.

## Abbreviations

BMD: Benchmark Dose
CINAC: Chronic Interstitial Nephritis in Agricultural Communities
CKD: Chronic Kidney Disease
CKDu: Chronic Kidney Disease of uncertain aetiology
CONTAM: Panel on Contaminants in the Food Chain
CV: Coefficient of Variation
EFSA: European Food Safety Agency
GFR: Glomerular Filtration Rate
GIS: Geographic Information Systems
ICP MS: Inductively Coupled Plasma Mass Spectrometry
IQ: Intelligence Quotient
LOQ: Limit of quantification
QC: Quality Control
RBF: Renal Blood Flow
SD: Standard Deviation
SEM: Standard Error of Mean
WHO: World Health Organization
WHO GEMS: World Health Organization General Environment Management System
